# Granulomatous colitis in a patient with metastatic melanoma under immunotherapy: a case report and literature review

**DOI:** 10.1186/s12876-021-01812-7

**Published:** 2021-05-19

**Authors:** Stratigoula Sakellariou, Dionysia N. Zouki, Dimitrios C. Ziogas, Despoina Pouloudi, Helen Gogas, Ioanna Delladetsima

**Affiliations:** 1 First Department of Pathology, Medical School, Laiko General Hospital, National and Kapodistrian University of Athens, 75, Mikras Asias street, 11527 Athens, Greece; 2 First Department of Internal Medicine, Laiko General Hospital School of Medicine, National and Kapodistrian University of Athens, Athens, Greece

**Keywords:** Immune checkpoint inhibitors-related colitis, Immune checkpoint inhibitors-related adverse events, Intracryptal granulomas, Granulomatous colitis, Ipilimumab-related granulomas

## Abstract

**Background:**

Immune checkpoint inhibitors (ICPIs) have changed the way advanced malignancies are currently confronted, improving cancer patients’ outcomes but also generating distinct immune-related (ir) adverse events. ICPIs-induced colitis is a common complication showing different clinical and histological manifestations. In the literature review, 14 cases with ICPIs related colon granulomas have been reported in 5 studies with either limited or unavailable information regarding histology. Granulomatous reactions can be mistakenly perceived as disease recurrence or progression. Better understanding and identification of this infrequent histological display can help to avoid misdiagnosis and mismanagement.

**Case presentation:**

A 63-year-old female patient with metastatic melanoma was admitted to the hospital with symptoms of nausea, persistent diarrhea and shivering fever under consecutive treatments with ICPIs, initially pembrolizumab and subsequently ipilimumab. Sigmoidoscopy was performed revealing mucosal edema, hyperemia and erosions of the rectum and sigmoid colon. Histological evaluation of sigmoid colon mucosa biopsies revealed an unusual colitis pattern characterized by multiple intracryptal granulomas attributed to ICPIs therapy. Steroids were administered and the patient recovered. ICPIs treatment was discontinued. The patient was subsequently treated with chemotherapy but follow up radiology showed disease progression. A re-challenge with another ICPI regimen was decided and the patient is currently under immunotherapy with stable disease regarding melanoma status and without any sign of colitis recurrence.

**Conclusions:**

The present report provides detailed histological description of a distinctive ICPIs-induced granulomatous colitis and highlights the need for awareness of the distinct adverse events and reaction patterns in the context of immunotherapy.

## Background

Over the past decade, immune checkpoint inhibitors (ICPIs) have rapidly evolved into a breakthrough oncological treatment, which changed the natural course of many different cancers and improved patients’ survival. Ipilimumab, a monoclonal antibody that blocks the cytotoxic T lymphocyte-associated antigen 4 (CTLA4) was the first agent approved for the treatment of late-stage melanoma. Subsequently two other ICPIs acting against PD1 receptor (programmed death receptor 1), pembrolizumab and nivolumab, received approval for advanced melanoma. Currently, an anti-PD1 regimen is the preferred immunotherapy with ipilimumab being administrated in combination with nivolumab or as second-line option [1].

Despite their impressive anticancer effects, ICPIs are also responsible for some serious immune-related adverse events (irAEs) affecting multiple organs such as skin, gastrointestinal (GI) tract, liver, lungs, lymph nodes, nervous and endocrine systems. Among irAEs, colitis is a very common immune-mediated complication requiring immediate clinical and histological evaluation, since severe cases can be life-threatening [2]. GI toxicity is more frequently reported in anti-CTLA4 administration, presenting with diarrhea of any grade in 27–54% of the patients [3]. The diagnosis of immune-related (ir) colitis is challenging as clinical manifestation is often atypical and non-specific mimicking other pathologic conditions such as intestinal bacterial infection or medication-associated diarrhea. The comprehensive approach of ir-colitis encompasses endoscopic and histological evaluation of colon mucosa. Typical colonoscopy findings are erythema, erosions, loss of vascular pattern and ulcerations, while in few cases mucosa may appear totally normal [3]. Histology reveals different patterns of injury, while granulomas have rarely been mentioned.

Herein, we describe a case of a female patient with metastatic melanoma under consecutive treatments with ICPIs, who developed a distinctive granulomatous colitis manifested by multiple intracryptal granulomas attributed to ICPIs therapy. A review of all cases with ICPIs related colon granulomas reported in the literature was also performed.

## Case presentation

A 63-year-old Caucasian female patient with no past medical history was diagnosed in 2014 with cutaneous melanoma on her right heel, Breslow thickness 8.5 mm, Clark level V, mitotic figures 10/mm^2^. Following primary diagnosis, she underwent wide local excision with sentinel lymph node dissection that was negative for residual disease. She subsequently received adjuvant high dose interferon (5 days/week for 4 weeks), according to approved guidelines at the time. Three years later, whole body computerized tomography (CT) scanning revealed a lesion of 45 mm in the lower lobe of the left lung. Patient underwent lobectomy and histology confirmed metastatic melanoma, BRAF wild type on molecular analysis. At that point, no adjuvant treatment was provided. A year later, CT restaging revealed several pulmonary nodules scattered on both lungs, enlarged lymph nodes in the left hilum and gastroesophageal area. Given melanoma relapse, the patient received pembrolizumab intravenously (iv), 200 mg flat dose every 3 weeks, without any immune-related complications except of a mild elevation of transaminases. Three months post pembrolizumab initiation, lesions and lymphadenopathy were increased in size and therefore anti-PD1 was discontinued. The patient was scheduled to receive 4 cycles of iv ipilimumab, 3 mg/kg every 3 weeks, as second-line treatment. Approximately 2 weeks after the second administration of ipilimumab, she complained of nausea, persistent diarrhea and shivering fever and was admitted to our clinic for further evaluation and management. On physical examination, fever reached 38 °C and her abdomen was distended with hyperactive bowel sounds. The white blood cell count was 6.48 × 10^9^/L with 71.3% neutrophils; C-reactive protein (135 mg/L, normal value < 5 mg/L) and lactate dehydrogenase (379U/L, normal range: 135–215 U/L) were increased while serum albumin was low (27.5 g/L, normal range: 35–50); transaminases and cholestatic enzymes were elevated (AST: 56 U/L, ALT: 45 U/L, GGT: 307 U/L, ALP: 170 U/L) with normal bilirubin (0.74 mg/dl, normal range: 0.3–1.2 mg/dl). Thyroid-stimulating hormone was increased (17.83 mU/L, normal range: 0.27–4.5 mU/L)] giving the suspicion for immune-mediated thyroiditis with the rest of serum endocrine parameters being normal [adrenocorticotropic hormone = 35 pg/ml (normal range: 10–65 pg/ml) and cortisol = 221.9 mmol/L (normal range: 173.6–505 mmol/L)]. Approaching her as an immunocompromised case, wide-range antibiotics were empirically delivered without resolution of her symptoms. Stool cultures for bacteria, ova and parasites, Clostridium difficile toxins (A and B), as well as polymerase chain reaction (PCR) for cytomegalovirus (CMV) were all negative. Abdominal CT scan was negative for visceral metastasis showing thickening of the large bowel wall, a finding supportive of colonic inflammation. The patient underwent sigmoidoscopy that revealed mucosal edema, hyperemia and erosions of the rectum and sigmoid colon. Representative biopsies were obtained for histological evaluation.

### Histological findings

**Fig. 1:**
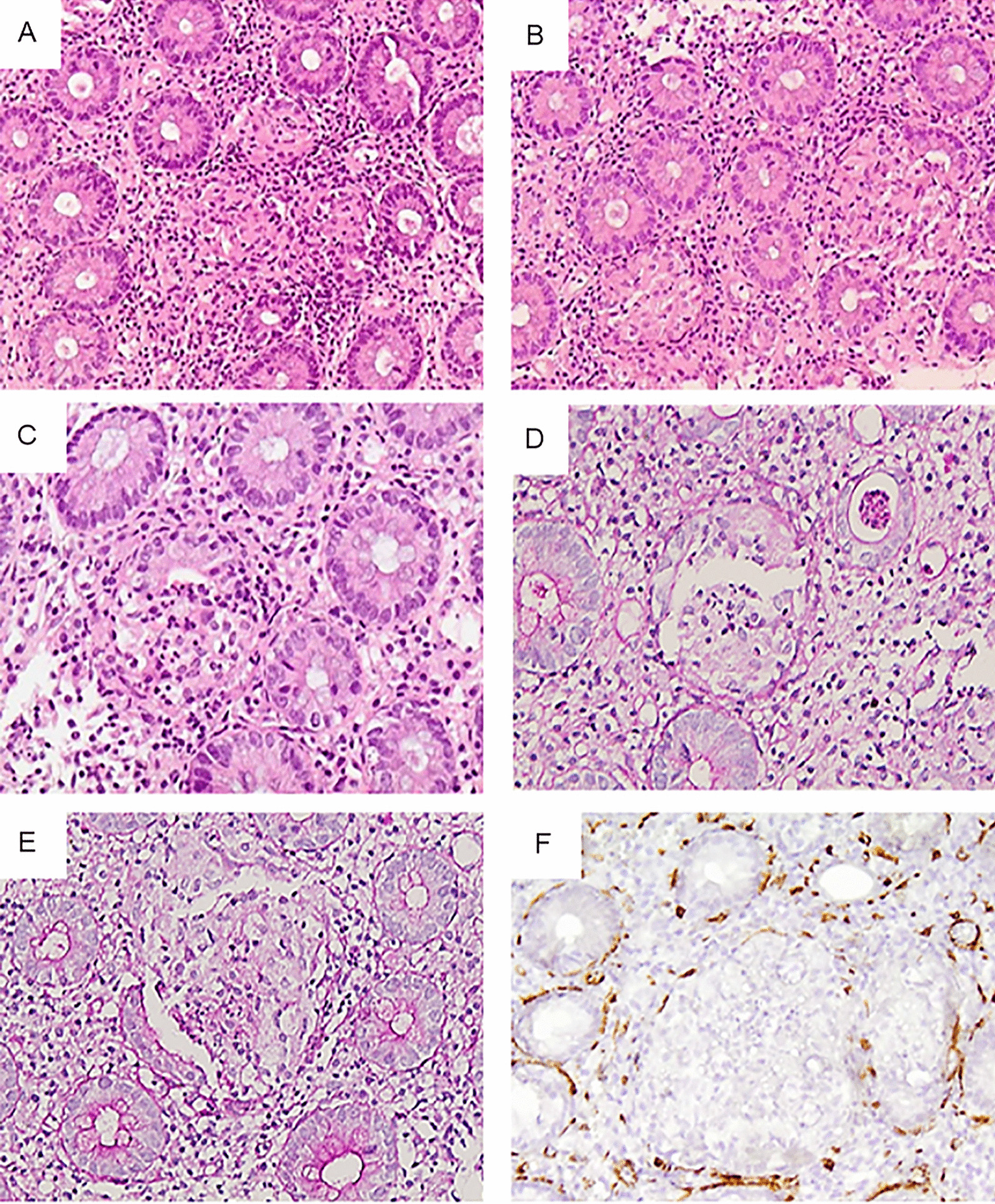
Intracryptal granulomas **a, b** Total and partial crypt wall destruction (H/EX100). **c**, **d** Partial destruction of the crypt epithelium and derangement but not total disruption of the basement membrane (H/EX200, PASX200). **e** Partial crypt wall destruction including epithelial lining and basement membrane (PASX200). **f** Attenuation of the pericryptal myofibroblasts (SMAX200)

Histological examination revealed multiple small, non-necrotizing epithelioid granulomas within the crypts with partial or complete destruction of the crypt epithelium and derangement but not total disruption of the basement membrane, depicted in Periodic Acid-Schiff (PAS) histochemical stain. Some intracryptal granulomas were accompanied by rupture of the crypt wall and pericryptal expansion (Fig. 1a–e). Active inflammation was also present characterized by moderate lamina propria lymphoplasmacytic infiltrations, focal cryptitis and rare crypt abscesses. Basal plasmacytosis, intraepithelial lymphocytes or crypt architectural distortions were not apparent while neutrophils and eosinophils were scarce. Few apoptotic bodies were occasionally seen at the crypt base. Histochemical PAS and Ziehl–Neelsen stains were negative for fungi and acid-fast bacteria respectively.

Immunohistochemistry was performed on 4 μm-thick sections using Dako Envision Flex system (Dako, Glostrup, Denmark). The following antibodies were applied: CD20 (Dako, clone L26, mouse monoclonal, 1:700 dilution, Glostrup, Denmark), CD3 (Dako, rabbit polyclonal, 1:50 dilution), CD4 (Dako, clone 4B12, mouse monoclonal, 1:50), CD8 (Dako, clone C8/144B, mouse monoclonal, 1:200 dilution) and SMA (Dako, clone 1A4, mouse monoclonal, 1:800 dilution). Based on the above immunostains, the vast majority of lymphocytes were T cells (CD3 +) predominately CD4+. SMA highlighted the attenuation of the pericryptal myofibroblasts (Fig. 1f).

In view of the patient’s clinical history, the lesion was reported as granulomatous colitis probably induced by ipilimumab.

### Management after ir-colitis

Anti-CTLA4 was discontinued and iv prednisone at a dose of 75 mg/d was initiated with immediate clinical improvement of the diarrheic syndrome. The patient was subsequently treated with chemotherapy with no reported serious adverse events till September 2019, when radiology showed new disease progression. A re-challenge with another anti-PD1 agent, namely nivolumab, was decided, and currently the patient is under immunotherapy with stable disease regarding melanoma status and without any sign of colitis recurrence.

### Literature review

**Fig. 2:**
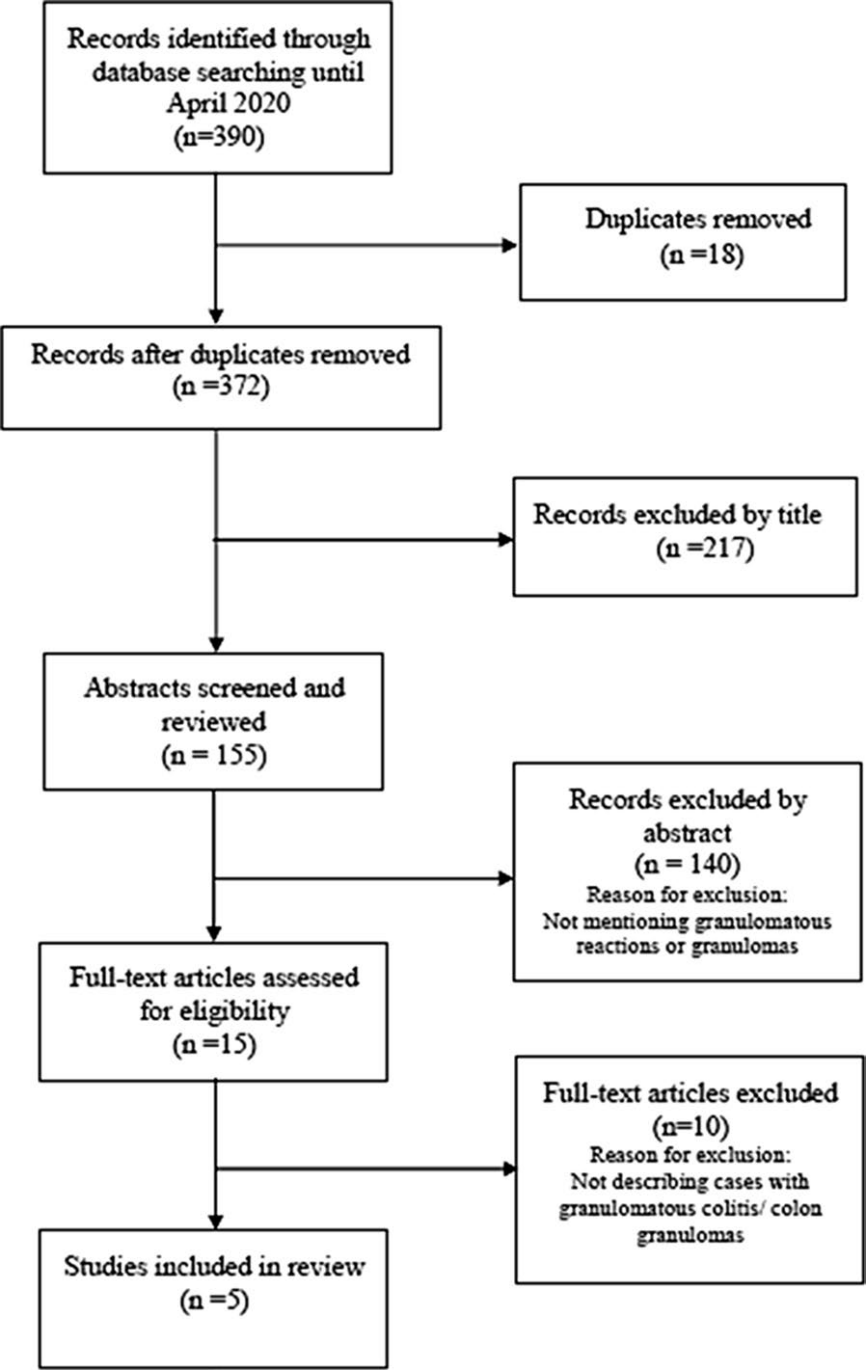
Flow diagram of the performed systematic review

Running through the literature on PubMed and using the following terms (CTLA4, PD1/PD-L1, ipilimumab, pembrolizumab, nivolumab, immune-related adverse events, granulomatous reactions, granulomatous colitis and colon granulomas), we ended up to 5 studies that described in total 14 cases with immunotherapy related colon granulomas. A flow diagram of the performed systematic review is illustrated in Fig. 2. In these cases, melanoma was commonly the underlying cancer treated with ICPIs, while the median time to colitis onset exceeded 30 days with one exception of ir-colitis presentation 27 days after the initiation of a combination immunotherapy.

Of the 14 reported cases of colitis with granuloma formation, 1 was associated with PD1 inhibitor [4], 2 with nivolumab/ipilimumab combination [5], 3 with an anti-PD1 regimen without being clearly mentioned if the patients were receiving also ipilimumab [6], and 5 with anti-CTLA4 treatment [5, 7]. For the remaining 3 cases it is not clear whether patients received ipilimumab alone or in combination with melanoma peptide vaccines to enhance host’s immunization [8], therefore granuloma formation could be related to ipilimumab, to vaccination or both. Table 1 summarizes the data of the studies reporting colon granulomas in patients under ICPIs.

**Table 1:** Summary of studies reporting colon granulomas in patients under ICPIs

Study	ICPIs	Malignancy	Patients with colon biopsies	Median time from drug initiation to GI symptoms onset	Colonoscopy findings	Patients with granuloma(s)	Granuloma features
Miyahara et al. [4]	anti-PD1 (Nivo or Pembro)	LC, PG	7	155 days (nivo)	Rough and friable mucosa, redness and erosions	1	NR
				138 days (pembro)			
Geukes Foppen et al. [5]	Ipi or anti-PD1 or Ipi + anti-PD1 or Ipi + RFA	MM, NSCLC	90	33 days (ipi)	Ulcers, diffuse inflammation, mucosa redness friability	5	Granulomas either in lamina propria or in submucosa
				84 days (anti-PD1)			
				27 days (Ipi + anti PD1)			
Gonzalez et al. [6]	anti-PD1 (Nivo or Pembro) or anti-PD1 + Ipi	MM, LC, UC, OSCC	17	84 days	Normal mucosa, mild colitis, severe colitis (diffuse or patchy erosions, friability)	3	Granulomasassociated with ruptured crypts
Marthey et al. [7]	Ipi	MM, NSCLC	27	34 days	Erythema, erosions, ulceration	2	Superficial granulomas
Beck et al. [8]	Ipi or Ipi + ΜΜ vaccines	MM, RCC	40	NR	Erythema, ulceration	3	NR

*ICPIs* immune checkpoint inhibitors, *Nivo* nivolumab, *Pembro* pembrolizumab *Ipi* ipilimumab, *RFA* radiofrequency ablation, *MM* metastatic melanoma, *LC* lung cancer, *UC* urothelial carcinoma, *OSCC* oral squamous cell carcinoma, *PG* pharyngeal cancer, *NSCLC* non-small cell lung cancer, *RCC* renal cell carcinoma, *NR* not reported, *GI* gastrointestinal

Apart from colon, granulomatous/sarcoid-like reactions (G/SLR) have also been described in different organs of cancer patients treated with ICPIs. Table 2 presents a synopsis of published series with 3 or more patients under immunotherapy reporting at least one case of ICPIs-associated G/SLR in organs other than GI tract [9–21]. Lungs, lymph nodes and skin were the main tissues with granuloma formation, while melanoma was the main underlying malignancy.

**Table 2:** Cases with ICPIs-associated granulomatous/sarcoid-like reactions in organs other than GI tract

Source	ICPIs used	Affected organ(s)	Malignancy	Patients with G/SLR/total patients
Johncilla et al. [9]	Ipi or Ipi + Nivo	Liver	MM	4/11
Belliere et al. [10]	Nivo	Kidneys	LC	1/3
Firwana et al. [11]	Ipi or Pembro	Spleen, LN, Skin	CC, MM	3/3
Cousin et al. [12]	Pembro	LN, Lung	LM	1/10*
Tetzlaff et al. [13]	Ipi or Pembro	Skin, LN	MM	3/3
De Martin et al. [14]	Ipi + Nivo or Ipi or Pembro	Liver	MM, BC	7/16
Faviez et al. [15]	Ipi + Nivo or Durva	Lung, LN, Spleen	LC, MM	3/3
Kubicki et al. [16]	Ipi or Ipi + BRAF inh	Skin	MM	3/3
Wang et al. [17]	Pembro	Skin, Lung	MM	1/17
Larsen et al. [18]	Pembro or Nivo	Lung	SqCC of skin, MCC, LC	3/9
Rodriguez et al. [19]	Ipi or Nivo or Ipi + Nivo or Ipi + Pembro	LN	MM	5/5
Zen et al. [20]	Atezo	Liver	LC	2/10
Chorti et al. [21]	Nivo or Ipi + Nivo	LN, Lung, Skin, Bone	MM	8/45

The patient indicated with an asterisk (*) was receiving also cyclophosphamide orally

*ICPIs* immune checkpoint inhibitors, *G/SLR* granulomatous/sarcoid-like reactions, *GI* gastrointestinal, *Ipi* ipilimumab, *Nivo* nivolumab, *Pembro* pembrolizumab, *Durva* durvalumab, *Atezo* atezolizumab, *inh* inhibitors, *LN* lymph nodes, *MM* malignant melanoma, *LC* lung cancer, *CC* colon cancer, *LM* leiomyosarcoma, *BC* bladder carcinoma, *SqCC* squamous cell carcinoma, *MCC* Merkel cell carcinoma

## Discussion and conclusions

ICPIs have transformed the therapeutic strategy for many malignancies and physicians have to face various aspects of their widespread oncological implication in everyday clinical practice. Among them, ir-toxicity is of major importance since it requires immediate recognition and treatment. In case of ir-colitis, endoscopy with biopsy collection is the initial fundamental step in the diagnostic work-up followed by a careful histological evaluation.

Histological features of anti-CTLA4 and anti-PD1-induced colitis overlap significantly. According to a recent review [22] four distinct histological patterns can be attributed to ipilimumab-induced colitis: active colitis, active colitis with prominent epithelial apoptosis, chronic active colitis mimicking idiopathic inflammatory bowel diseases (IBD) and lymphocytic colitis. Lamina propria infiltration by lymphocytes, plasma cells and a varying number of neutrophils and eosinophils is constantly present. Cryptitis and crypt micro-abscesses are also common findings. Increased epithelial apoptotic bodies at the base of the crypts is characteristic feature of the “active colitis with prominent epithelial apoptosis”. An IBD-like pattern with signs of chronicity such as basal plasmacytosis, significant crypt architectural distortion and Paneth cell metaplasia in the distal colon seems to evolve if ipilimumab-colitis is left untreated, immunosuppressive treatment proves to be ineffective or after recurrent episodes of colitis. A histological subtype mimicking lymphocytic colitis with increased intraepithelial lymphocytes, surface epithelial injury and minimal neutrophilic infiltration has also been described [22, 23]. In a previous study from our department [24] ipilimumab-related inflammation always involved the sigmoid colon showing pathologic features mostly resembling to IBD. Anti-PD1-associated colitis usually appears as active colitis showing cryptitis and neutrophilic crypt abscesses accompanied by increased apoptosis and crypt atrophy/dropout. Another histological pattern is “lymphocytic colitis” similar to the one observed in ipilimumab-related injury, while recurrent anti-PD1 colitis can result in IBD-like chronic active colitis [23, 25]. Few cases of ICPIs-induced collagenous colitis have also been reported [26–28]. Concerning lymphocytic subsets, T cell population prevails in ICPIs-associated colitis. CD8 + T-cells predominate in the lamina propria and epithelium of anti-PD1 related colitis, whereas CD4 + T-cells are more numerous in anti-CTLA4-induced colitis [24, 29, 30]. Our case does not fulfill the histological features of the above-mentioned colitis subtypes and seems to represent a distinctive colitis pattern determined by intracryptal granulomas.

In most of literature cases showing granuloma formation after immunotherapy, histological details are not available. Geukes Foppen et al. [5] report that granulomas were found mainly in the lamina propria of colon mucosa and rarely in the submucosa, while in another study [6] granulomas were seen in a minority of the examined biopsies in relation to ruptured crypts and were considered secondary to crypt damage. Our case is differentiated by the presence of multiple, minute, epithelioid granulomas, the majority confined within the crypt limits, as shown by the preservation of the basement membrane and subepithelial myofibroblasts. These findings favor intracryptal formation as initial reaction followed by disruption of the crypt wall. Considering that granulomas were the prevailing finding, the term granulomatous colitis is justified.

To support the diagnosis of ICPIs-induced colitis, we excluded a history of inflammatory bowel disease as well as infections that can be manifested as granulomatous colitis. The onset of diarrhea after ICPIs exposure and the resolution of symptoms soon after steroid administration were in favor of our diagnosis. Given the PD1 inhibition with pembrolizumab before ipilimumab administration, we cannot be sure whether the clinical syndrome and the granulomatous inflammation was an anti-CTLA4 or an anti-PD1 side effect or a consequence of their sequencing. However, there are some data supporting the causality of ipilimumab. The onset of GI symptoms following ipilimumab administration (e.g. two weeks after the second dose and 5 after its initiation) is in agreement with the time-point of irAE presentation according to published evidence, which suggest that ir-colitis may occur at any time during 1–10 infusions of anti-CTLA4 [3]. Moreover, no colitis recurrence was observed for more than 4 months after re-induction of anti-PD1 treatment and finally the predominance of CD4 + T lymphocytes also favor the implication of anti-CTLA4  agent.

As far as other parts of GI tract are concerned, 2 cases of anti-CTLA4-related granulomatous gastritis and 1 of duodenitis have been reported, unaccompanied by histological description [7]. Although rare in GI, granulomatous/sarcoid-like reaction (G/SLR) associated with anti-PD1/PD-L1 or anti-CTLA4 antibodies is an already recognized immunotherapy-induced toxicity that can affect different organs. Better understanding and identification of this infrequent histological display can help to avoid misdiagnosis and mismanagement, since G/SLR can be erroneously perceived as disease recurrence or progression [31]. It has also been suggested that the formation of granulomas itself may not represent an irAE but rather a part of antitumor immune response with self-limited potential not requiring steroid treatment [32, 33].

The exact mechanism of granulomas formation in sarcoidosis, Crohn’s disease and similar granulomatous reactions is currently unknown. There are indications of a T cell-mediated phenomenon involving T-helper (Th) and T-regulatory (Tregs) cells. Uncontrolled activity of Th1 cells with overproduction of cytokines together with already present macrophages seem to play a crucial role in the development of granulomas [34]. Two recently published studies identified significant up-regulation of mucosal expression of TGF-α and IFN-γ in anti-CTLA4-induced colitis supporting the enrollment of IFN-γ in pathogenesis [24, 30]. Furthermore, decreased CTLA4 expression on Tregs has been reported in patients with sarcoidosis permitting incrimination of CTLA4 and Tregs function defects in granuloma formation in autoinflammatory diseases [35]. Based on the latter assumption, the suppression of Tregs by anti-CTLA4 could be pathogenetically involved in granulomatous reaction. One should not overlook the fact that in our case granulomas displayed most often intracryptal topography favoring infectious or non-infectious particles of the luminal content as foreign triggering factors. It could also be speculated that crypt epithelial lesions may also present an initial immune-mediated damage aggravated by the following granulomatous reaction.

This report presents a rare case of ICPIs-induced granulomatous colitis and highlights the need for awareness of the distinct adverse events and reaction patterns in the context of immunotherapy, in order to avoid misdiagnosis. It is crucial for the medical community not only to be vigilant for the reported iRAEs and their management, but also for novel reactions mediated by these revolutionary drugs.

## Data Availability

The datasets used and/or analyzed during this study are included in this paper and shall be available from the corresponding author upon request.

## Abbreviations

ICPIs: Immune Checkpoint Inhibitors
Ir: Immune-related
CTLA4: Cytotoxic T-lymphocyte—associated Antigen 4
PD-1: Programmed—death receptor 1
PD-L1: Programmed death ligand 1
IrAEs: Immune—related adverse events
GI: Gastrointestinal
CT: Computerized tomography
BRAF: V-Raf murine sarcoma viral oncogene homolog B
Iv: Intravenously
PCR: Polymerase chain reaction
CMV: Cytomegalovirus
PAS: Periodic acid—Schiff
CD: Cluster of differentiation
SMA: Smooth muscle actin
G/SLR: Granulomatous/sarcoid—like reactions
IBD: Inflammatory bowel diseases
Th: T helper
Tregs: T regulatory
TGFα: Transforming growth factor alpha
IFN-γ: Interferon gamma
